# Long-Term Toxicity and Efficacy of Intensity-Modulated Radiation Therapy in Cervical Cancers: Experience of a Cancer Hospital in Pakistan

**DOI:** 10.1200/GO.20.00169

**Published:** 2020-10-28

**Authors:** Muhammad Atif Mansha, Tabinda Sadaf, Asmara Waheed, Amna Munawar, Asma Rashid, Samreen Javed Chaudry

**Affiliations:** ^1^ Clinical and Radiation Oncology Department, Shaukat Khanum Memorial Cancer Hospital and Research Centre, Lahore, Pakistan

## Abstract

**PURPOSE:**

To report the chronic toxicity and disease outcomes attributable to intensity-modulated radiation therapy (IMRT) in patients with cervical cancer.

**METHODS AND MATERIALS:**

Between January 2014 and December 2018, a retrospective review of medical records of patients with cervical cancer who received radiation therapy with IMRT was performed. Disease and treatment-related details were documented. Follow-up notes were reviewed, and severity of late toxicities was recorded. Overall survival (OS) and disease-free survival (DFS) at 3 years were estimated.

**RESULTS:**

A total of 222 patients’ records were reviewed. Mean age was 50.7 years. Median follow-up duration was 33 months (range, 2-70 months). The most common toxicity was vaginal stricture (grade 2, n = 59, 26.6%; grade 3, n = 4, 1.80%), followed by proctitis (grade 2, n = 24; 10.8%; grade 3, n = 7; 3.20%). Seven patients (grade 2, n = 5, 2.3%; grade 3, n = 2; 0.90%) developed cystitis, and only 5 (grade 2; 2.3%) were found to have colitis. None of the patients had grade 4 or grade 5 toxicities. There was a significant difference in late complications in patients with nodal disease or those who underwent prior surgery (*P* < .05). Three-year OS and DFS rates were 79.7% and 81.9%, respectively. Patients with tumor size > 5 cm and those with pelvic lymph node metastasis had poor survival rates (*P* < .05).

**CONCLUSION:**

IMRT is an effective and well-tolerated technique that should be considered in patients with lymph node disease and in postoperative patients. There is an inverse relationship between tumor size and nodal involvement with respect to OS and DFS.

## INTRODUCTION

Cervical cancer is an aggressive disease and remains the most common gynecologic malignancy worldwide. It is the third most common cancer for women in Pakistan, after breast and oral cavity cancer.^1^ Multiple therapeutic modalities have been used in the management of this ominous disease. Since the early 1900s, radiation therapy (RT) has been used with curative intent in the treatment of cervical cancers. Literature has shown that surgery or RT are equally effective in terms of curing the disease in the early stages.^2,3^ At present, the standard treatment approach for locally advanced cervical cancers is concurrent chemoradiation.^4^

**Context**

**Key Objective**
Does intensity-modulated radiation therapy (IMRT) reduce long-term toxicity and improve overall survival (OS) and disease-free survival (DFS) in patients with cervical cancer?
**Knowledge Generated**
There was a significant difference in late grade 2 or higher radiation-induced toxicities in patients with pelvic or para-aortic lymph node metastasis and in those who underwent prior surgery when treated with IMRT. IMRT significantly affects OS and DFS in patients with a primary tumor size > 5 cm or with pelvic lymph node involvement.
**Relevance**
IMRT should be considered a preferred radiation modality in the postoperative setting and in patients with bulky tumors and nodal disease.

Traditionally, RT has been delivered to the whole pelvis via conventional methods with either four-field box technique or two parallel opposed anteroposterior fields. These approaches deliver homogenous doses of radiation to the entire pelvis exposing both tumor and normal organs to high radiation doses, which subsequently results in considerable toxicity.^5^

The transition from conventional RT to 3-dimensional conformal RT (3D-CRT) has allowed for use of an increased number of radiation beams that are shaped to conform to the target volume.^6^ Intensity-modulated RT (IMRT) is an advanced form of 3D-CRT that achieves an even greater conformity by optimally modulating the individual beamlets that make up a radiation beam. IMRT relies on computer control capabilities generating dose distributions conforming much more closely to the target volumes, avoiding critical normal structures and reducing treatment-related toxicity.^7^

Long-term toxicities are a potential cause of late morbidity persisting for many years and have been understated in the contemporary literature. A systematic review of randomized trials highlighting the incidence of acute and late toxicity in the treatment of patients with cervical cancer showed that only 8 of 19 trials described long-term toxicity and without any statistical significance.^8^ But recently, with an increased emphasis on long-term quality of life, the importance of late sequelae is being increasingly recognized. Advance conformal techniques have led to a significant reduction in late toxicities affecting quality of life and also potentially improved the disease-related outcomes.^9^

Pakistan is still in the infancy of technologic development. At Shaukat Khanum Memorial Cancer Hospital and Research Centre (SKMCH&RC), we began to treat patients with cervical cancer with IMRT in 2014. Most of our patients have completed a follow-up of more than 30 months. The purpose of this study was to report late toxicity and survival outcomes of patients treated with IMRT in our center. The data collected during this study will enable us to compare our results with contemporary international literature and will lay a foundation to guide amendments in current protocols. Such indigenous statistics will help us to amass a body of evidence and devise better treatment planning strategies for our patients with cervical cancer.

## METHODS AND MATERIALS

A retrospective review of electronic medical records of patients with cervical cancer who were scheduled for RT to the pelvis at SKMCH&RC between January 1, 2014, and December 31, 2018, was performed. All the patients received RT via the IMRT technique during this period.

A complete history and physical examination, including gynecologic examination, tissue biopsy for histopathologic diagnosis, magnetic resonance imaging (MRI) of the pelvis, and computed tomography (CT) of the chest and abdomen, were performed to investigate the extent of the disease. Any lymph node with a diameter > 1 cm in the short axis was considered metastatic. The disease was staged using 2009 International Federation of Gynecology and Obstetrics (FIGO) staging for cervical cancers. All patients were discussed in a multidisciplinary gynecologic cancer conference before starting treatment.

Patients were simulated in the supine position with their arms above their heads. Vac-Lok cushions and a minimum of three radiopaque markers were used to stabilize the position of the patients. Patients were advised to drink 300-500 mL of water or clear fluid 30 minutes before acquiring planning CT images. A CT scan was performed with intravenous contrast at a slice thickness of 3 mm.

The tumor and target volumes were contoured per institutional guidelines. Gross tumor volume (GTV) was marked by delineating the primary tumor in CT slices. GTV-nodal was contoured for any pelvic or para-aortic lymph node measuring ≥ 1 cm in the short axis. Clinical target volume (CTV) was contoured by including GTV and the uterus, cervix, parametria, and part of the vagina 2 cm distal to GTV. When disease involved the vagina, the upper half of the vagina was included in the CTV. CTV-nodal comprised GTV-nodal together with a 7-mm margin to common iliacs, external iliacs till femoral heads, and internal iliacs along with their branches (obturator and hypogastric) terminating in paravaginal tissues at the level of vaginal cuff. Presacral lymph nodes were also included in CTV-nodal by contouring the lymph node region anterior to the first and second sacral vertebrae. For patients with para-aortic lymph node involvement, CTV-nodal was extended 2 cm above the involved nodes. Planning target volume (PTV) was created by giving a margin of 10 mm to CTV.

The planning dose was 45.0-50.4 Gy in 25-28 fractions to the PTV and an additional boost of 9-16 Gy was given in case of gross disease after 50.4 Gy. The plan was generated using five to seven coplanar beams, and dose was delivered with 6 megavoltage photons. Daily cone beam CT was used during the course of radiation for image guidance.

During the last week of external-beam RT, intracavitary brachytherapy was performed using remote after loading high-dose-rate (HDR) unit with iridium-192 source. A dose of 24 Gy in 4 fractions was delivered to high-risk CTV using a tandem and two ovoids under a CT-guided procedure. In patients who had hysterectomy, only ovoids were placed. The entire treatment was completed within 56 days.

Patients received concurrent chemotherapy with weekly cisplatin 40 mg/m^2^. For patients with compromised renal function or renal failure, carboplatin area under curve 2 or paclitaxel was given. Because of locally advanced disease and radiotherapy waiting times, patients with a tumor size > 5 cm or lymph nodes > 1 cm were given 2 cycles of neoadjuvant chemotherapy with carboplatin and paclitaxel.

Patients who underwent surgery before presenting at our center were considered for RT if they had inadequate surgery with residual disease left behind. Patients with a tumor size > 5 cm and histopathologically reported lymphovascular space invasion, deep one-third stromal invasion, microscopic parametrial or margin involvement, and lymph node disease were considered for adjuvant RT.

Patients were examined weekly during the course of radiation treatment, and once the treatment was completed, all the patients were evaluated after 6 weeks for any subjective complaints. The subsequent follow-up visit was scheduled 3 months after treatment completion, and a comprehensive gynecologic examination was performed. Before this visit, all the patients were advised to undergo MRI scanning of the pelvis. The clinical response assessed on physical examination and radiologic response evaluated with RECIST guidelines were recorded. Patients were then evaluated every 6 months for 2 years and then annually with clinical examination and MRI of the pelvis. Chronic toxicities were documented, and severity was graded according to Common Terminology Criteria for Adverse Events (CTCAE) version 4.^10^

Overall survival (OS) was defined as the length of time in months from the diagnosis of the disease until death from any cause. Disease-free survival (DFS) was defined as the length of time from the completion of RT until the disease relapsed. The relapse was documented as either local or distant. Diagnostic and therapeutic details were presented in the form of frequencies and percentages. Statistical inferences were drawn using χ^2^ test. The Kaplan-Meier method was used to estimate OS and DFS. Log-rank test was used to examine statistical significance. A *P* value < .05 was considered significant. Statistical Package for Social Sciences version 20 (SPSS, Chicago, IL) was used for statistical analysis.

## RESULTS

A total of 222 patients were treated with IMRT at SKMCH&RC. Mean age of the patients at presentation was 50.77 years. Of 222 patients, 121 (54.5%) had FIGO stage II disease. Most common histology was squamous cell carcinoma (n = 184; 82.9%) and majority of the tumors were of intermediate grade (n = 146; 65.8%). Regional lymph node metastasis was found in 126 patients (56.8%), with pelvic nodes involved in 111 (88.1%) and para-aortic nodal disease in 15 patients (11.9%). Additional details of disease are shown in Table 1.

**TABLE 1:**
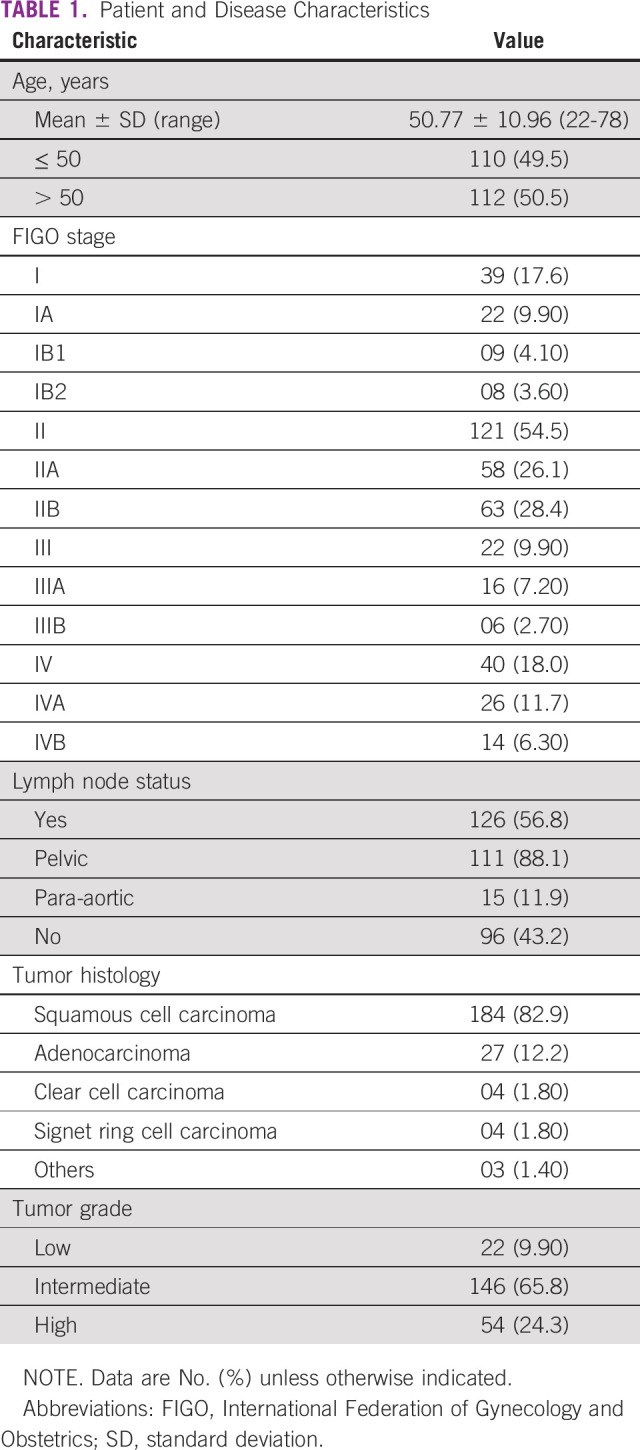
Patient and Disease Characteristics

Neoadjuvant chemotherapy was offered to 86 (38.7%) patients, whereas 68 (30.6%) patients underwent surgery before RT. Fifty-one patients (23%) received two cycles of neoadjuvant chemotherapy, whereas 27 (12.2%) received three courses. Six (2.7%) patients received only one course, and two patients were given four and five cycles each. There was a reduction in tumor size in 78 patients (91.8%) on clinical examination. Disease progression was observed in five patients (5.9%), and three patients (2.4%) had stable disease.

A radiation dose of 50.4 Gy in 28 fractions was given to 88 patients (39.6%), whereas 73 (32.9%) received 50.0 Gy in 25 fractions. In 17 patients (7.7%), 59.4 Gy was delivered in 33 fractions. Doses of 54.0 Gy in 30 fractions and 45.0 Gy in 25 fractions were given in nine (4.1%) and seven (3.2%) patients, respectively. A total of 13 patients (6.1%) received doses equal to or in excess of 60.0 Gy, with one patient receiving a maximum dose of 66.6 Gy in 31 fractions. Only four patients (1.9%) received doses < 45.0 Gy. All the fractions were delivered as part of a once-daily fractionation scheme, 5 days per week.

In conjunction to IMRT, HDR brachytherapy with iridium-192 radioactive isotope was given in 206 patients (92.8%). A dose of 24.0 Gy was delivered in four fractions to 191 patients (92.7%). The maximum dose of radiation given via brachytherapy was 28.0 Gy in four fractions (n = 1; 0.5%). Two fractions were delivered in 1 day, 6 hours apart, with the next two fractions delivered after a gap of 1 week. One patient (0.5%) received 21.0 Gy in three fractions. Thirteen patients (5.9%) did not complete a scheduled course of brachytherapy.

**FIG 1:**
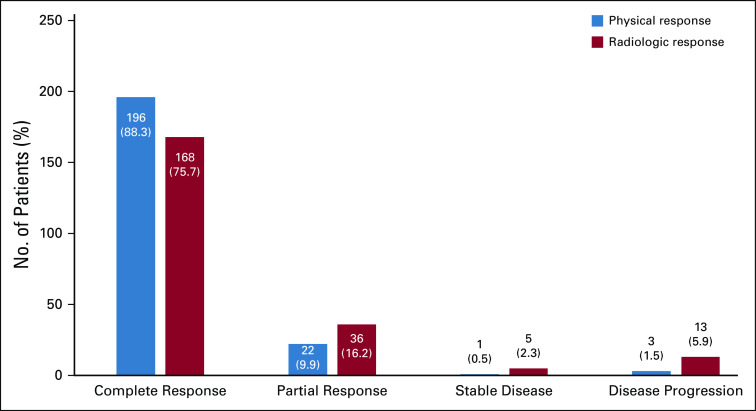
Frequency of different types of responses after completion of radiation therapy.

Concurrent chemotherapy was given in 212 patients (95.5%). Cisplatin was the most common drug used in the concomitant setting (n = 208; 98.1%), followed by carboplatin (n = 3; 1.41%) and paclitaxel (n = 1; 0.47%). A total of 157 patients (74%) received five cycles of concomitant chemotherapy. The radiation treatment was completed in a mean duration of 45 days, with a range of 6-70 days. The details of various treatment-related characteristics are shown in Table 2. Most of the patients had complete physical (n = 196; 88.3%) and complete radiologic response (n = 168; 75.7%) to the offered treatment. The details of physical and radiologic responses documented after 3 months of completion of treatment are shown in Figure 1.

**TABLE 2:**
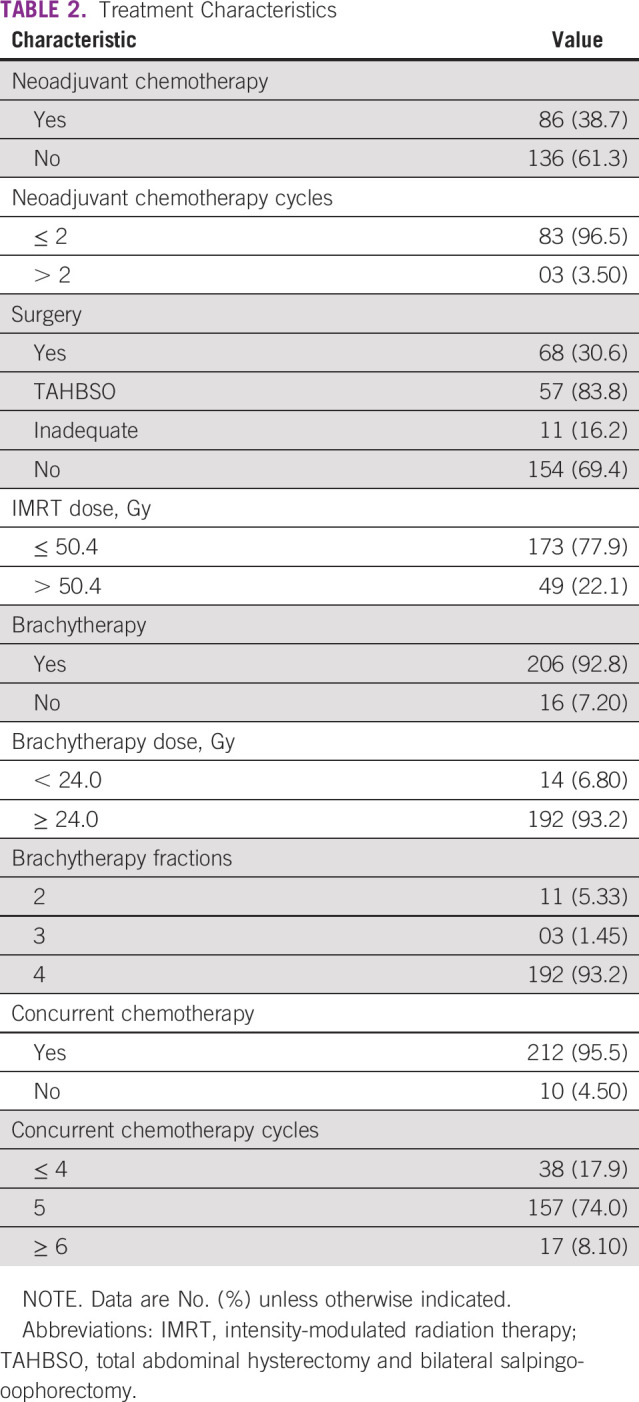
Treatment Characteristics

The median follow-up duration was 33 months (range, 2-70 months). Of 222 patients, 136 (61.3%) had grade 1 toxicity, whereas 86 (38.7%) developed grade 2 or greater toxicities. Vaginal stricture (27.6%) was found to be the most frequent toxicity among patients with grade 2 or greater toxicity, followed by proctitis (14%). There were no grade 4 or grade 5 toxicities reported. Details of different toxicities are shown in Table 3. Lymph node involvement, both pelvic and para-aortic, and surgery were the factors significantly affecting grade 2 or greater toxicities, as shown in Table 4.

**TABLE 3:**
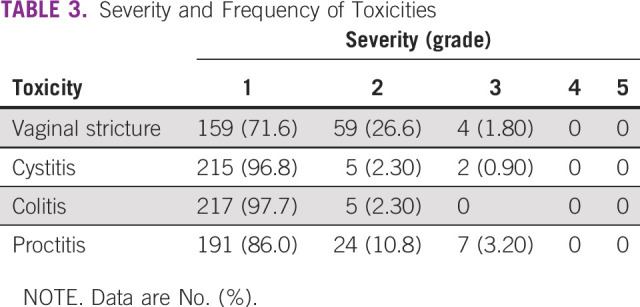
Severity and Frequency of Toxicities

**TABLE 4:**
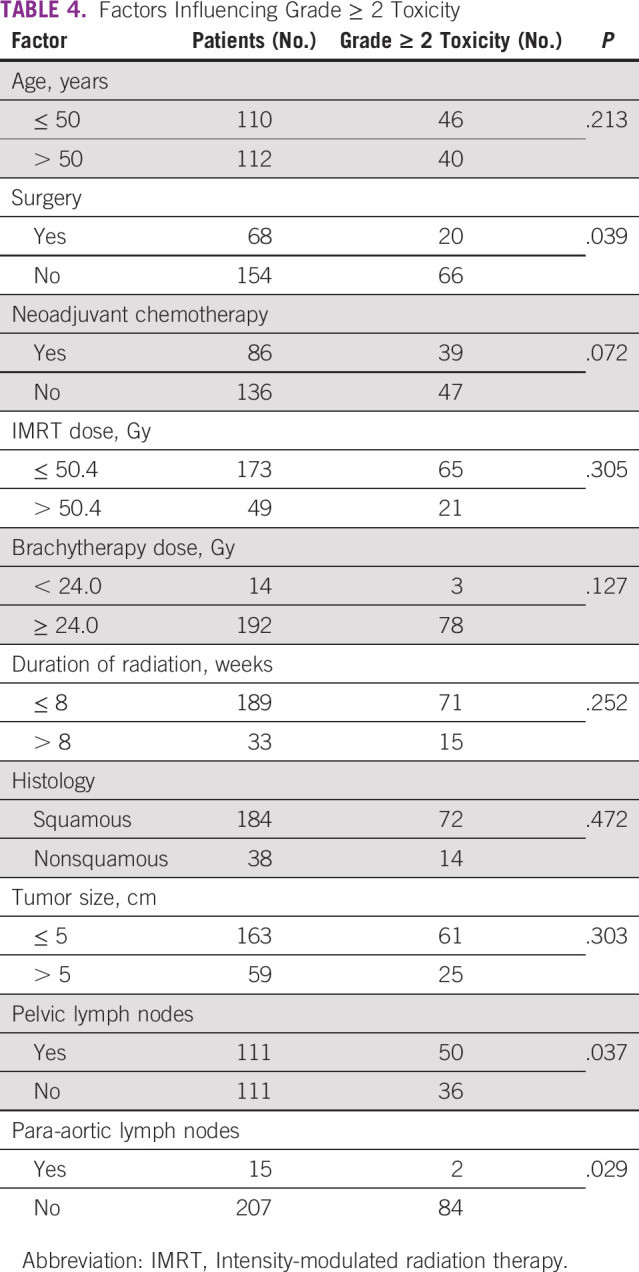
Factors Influencing Grade ≥ 2 Toxicity

**FIG 2:**
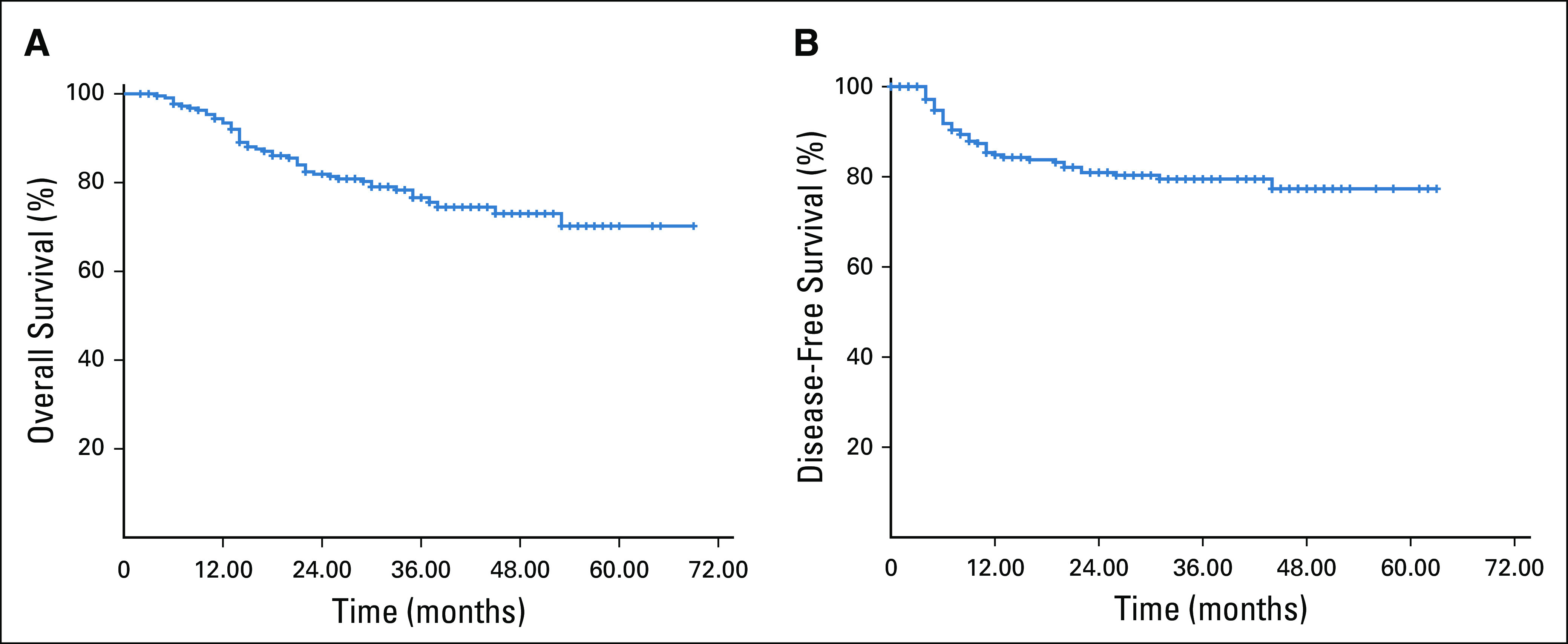
Kaplan-Meier curves showing (A) overall survival and (B) disease-free survival.

The mean OS was 55.57 months, and mean DFS was 51.88 months. Disease relapsed in 41 patients (18.5%), with local relapse in nine (22%) and distant relapse in 32 (78%). Tumor size > 5 cm and pelvic lymph node disease was associated with poor OS and DFS (Table 5). Three-year OS and DFS rates were 79.7% and 81.9%, respectively, as shown in Figure 2. The reason OS is slightly less than DFS is that most of the recurrences occurred within the first year after the completion of treatment and after that recurrences were stable.

**TABLE 5:**
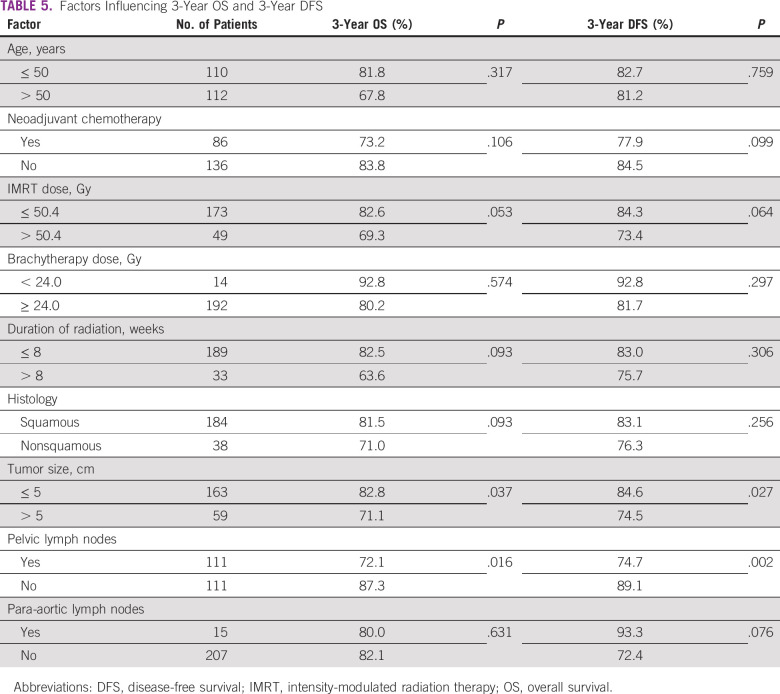
Factors Influencing 3-Year OS and 3-Year DFS

## DISCUSSION

There is a dearth of data regarding the use of IMRT for the treatment of cervical cancers in low- and middle-income countries (LMICs).^11-13^ Although there are a few reports from LMICs commenting on the occurrence of acute symptoms in patients receiving RT to the pelvis for gynecologic cancers, the literature highlighting chronic toxicities is scarce.^14,15^ Such acute reactions can be attributable to chemotherapeutic agents used in the concomitant setting with RT. The need to monitor for late effects mandates that the radiation oncologist stay engaged in long-term follow-up clinics. In the context of LMICs, it may not be feasible, but we overcame the challenge with the help of our dedicated and experienced team, respecting the patients’ values and perspectives about the disease and its treatment, ultimately fostering a bond leading to extraction of quality information from the patients and their families.

Mundt et al^16^ were the first ones to explore the use of IMRT in gynecologic malignancies. They reported a clinical series of 36 patients treated with IMRT and compared them with 30 historic controls treated with conventional RT. This study had a short median follow-up of 19.6 months for patients treated with IMRT. Chronic GI toxicity was much less in patients treated with IMRT (11.1% *v* 50%; *P* = .001). Patient age was the only factor significantly correlating with the incidence of late GI toxicity, with only 13.8% of patients aged ≤ 50 years developing toxicity compared with 40.5% among those aged > 50 years (*P* = .02). Mundt et al^16^ assessed all the patients with the help of a self-developed questionnaire consisting of a four-point toxicity grading scale, which might lead to a subjective bias. In our study, pelvic examinations under anesthesia and diagnostic interventions like colonoscopy or cystoscopy were performed by subject specialists in all the relevant patients to confirm the severity of toxicity.

Our study showed a significant difference in late toxicities in patients with nodal metastasis, with 45% of patients with pelvic nodal involvement developing grade 2 or more late complications. Consistent with our results, the incidence of grade 2 or more late toxicities reported by Lei et al in their patients was 42.5%.^17^ Interestingly, only 4.5% of our patients with pelvic nodal disease had grade 3 GI toxicity, which was in contrast to 8.5% observed by Lei. Another stark difference was an incidence of grade 2 genitourinary (GU) toxicity in patients with involved pelvic lymph nodes, with only 0.9% reported by Lei as compared with 2.7% shown in this study.

A retrospective study by Wang et al^18^ reported 3-year OS and DFS rates of 83% and 75% in patients receiving IMRT, consistent with our study (79.7% and 81.9%), respectively. They reported more grade 2 or more chronic GI toxicity in 16.4% patients, similar to our study, where 15.3% of patients had grade 2 or more late GI toxicity. But the rate of chronic GU toxicity was 30.6% in our patients in contrast to 11.3% reported by Wang et al.^18^ Interestingly, their data revealed that six patients developed rectovaginal or vesicovaginal fistulas, with none such complications reported in our patients.

It is still difficult to determine whether the use of IMRT results in better survival outcomes. A multi-institutional study evaluating the toxicity and survival outcomes after IMRT in 111 patients reported a 3-year OS rate of 78% and 3-year DFS rate of 69%.^19^ Recently, a meta-analysis of six studies conducted by Lin et al^20^ compared the efficacies and toxicities of IMRT with conventional RT for radical treatment of cervical cancers. They concluded there was no significant difference between 3-year OS and DFS, but toxicities were significantly reduced.^20^

There are certain shortcomings in our study. We were not able to carry out a head-to-head comparison of IMRT with conventional RT. This feat might not be possible with an increasing number of physicians and patients opting for IMRT as a preferred treatment modality. Therefore, it is difficult to draw an inference accrediting IMRT to a low incidence of late toxicity. A prospective randomized trial conducted in India comparing both radiation treatment modalities did not show any significant difference between OS and DFS. However, patients treated with IMRT were associated with less chronic GI toxicity with IMRT (13.6% *v* 50%; *P* = .011). The percentage of patients with grade 2 or more late GI toxicities reported in this study was 4.5%, much less than in our patients, where 15.3% of patients had chronic grade 2 or more GI toxicity.^21^ However, the sample size and follow-up time in this study was insufficient to draw any meaningful conclusion. Comparatively, there was a small nodal CTV-to-PTV margin, and no dose escalation or para-aortic RT was considered.

Critics can be of the opinion that most of the toxicity, vaginal stricture, or proctitis, may be influenced more by brachytherapy than by the external-beam component of treatment. We routinely advise our patients to use vaginal dilators and check their compliance on follow-up visits. Similarly, rectal doses are always kept within tolerance limits.

Almost all the previous studies used Radiation Therapy Oncology Group (RTOG) and European Organisation for Research and Treatment of Cancer (EORTC) toxicity criteria to grade late effects.^22^ On the contrary, we used CTCAE version 4, because it is more comprehensive and builds on the strengths of previous versions and a multimodal grading system for reporting late effects of cancer treatment.^10^ RTOG/EORTC toxicity criteria do not distinguish urinary and gynecologic toxicities as separate entities, using the broad label of GU toxicity. Similarly, the term small/large intestine is inclusive of both colitis and proctitis.

This report is consistent with the existing literature in acknowledging IMRT as a therapeutic modality resulting in fewer long-term complications in patients with cervical cancers. There is a need to further identify a subset of patients with cervical cancer who potentially benefit from IMRT in terms of survival outcomes. However, the definite advantage of reduced toxicity demonstrated in the current literature outweighs the equivocal survival benefit, and therefore, IMRT should be considered the standard technique for delivering RT.
